# The Diagnostic and Prognostic Role of Combined *p16* and *MTAP* Immunohistochemistry in Melanocytic Tumors of Uncertain Malignant Potential: A Comprehensive Review and Clinical Practice Analysis

**DOI:** 10.3390/ijms27020971

**Published:** 2026-01-19

**Authors:** Ludovica Pepe, Vincenzo Fiorentino, Cristina Pizzimenti, Maurizio Martini, Mariacarmela Santarpia, Antonina Fazio, Mario Vaccaro, Maria Lentini, Antonio Ieni

**Affiliations:** 1 Anatomic Pathology Unit, Department of Human Pathology in Adult and Developmental Age “Gaetano Barresi”, University of Messina, 98125 Messina, Italy; ludopepe97@gmail.com (L.P.); vincenzo.fiorentino@unime.it (V.F.); maurizio.martini@unime.it (M.M.); maria.lentini@unime.it (M.L.); 2 Anatomic Pathology Unit, Papardo Hospital, 98158 Messina, Italy; cristinapizzimenti86@gmail.com; 3 Medical Oncology Unit, Department of Human Pathology “G. Barresi”, University of Messina, 98122 Messina, Italy; mariacarmela.santarpia@unime.it; 4 Department of Human Pathology, Plastic Surgery Unit, University of Messina, Policlinico Hospital, 98122 Messina, Italy; tanifazio25@gmail.com; 5 Department of Clinical and Experimental Medicine, University of Messina, Via C. Valeria, 98125 Messina, Italy; mario.vaccaro@unime.it

**Keywords:** Melanocytic Tumors of Uncertain Malignant Potential (MELTUMP), p16 immunohistochemistry, MTAP (Methylthioadenosine phosphorylase), 9p21 locus, *CDKN2A*, diagnostic uncertainty, prognostic markers

## Abstract

Melanocytic Tumors of Uncertain Malignant Potential (MELTUMPs) remain among the most challenging entities in dermatopathology due to overlapping morphologic features and marked inter-observer variability. This comprehensive review critically assesses the diagnostic and potential prognostic significance of combining p16 and methylthioadenosine phosphorylase (MTAP) immunohistochemistry (IHC) as a practical surrogate for genomic alterations involving the 9p21 (*CDKN2A/MTAP*) locus. We analyzed the molecular underpinnings of the *CDKN2A/MTAP* axis and systematically reviewed existing literature to define an integrated IHC strategy for ambiguous melanocytic lesions. The combined use of p16, a sensitive marker of *CDKN2A* inactivation, and *MTAP*, a highly specific marker for homozygous 9p21 deletion, was assessed for its diagnostic complementarity and potential clinical utility. p16 IHC demonstrates high sensitivity but limited specificity due to heterogeneous staining in borderline lesions. In contrast, MTAP loss exhibits near-absolute specificity for *CDKN2A/MTAP* co-deletion, albeit with lower sensitivity. Concordant loss of both markers strongly supports melanoma or high-risk melanocytoma, while *MTAP* retention may predict responsiveness to adjuvant interferon therapy. Combined p16/MTAP IHC provides a synergistic, biologically grounded approach that refines diagnostic accuracy in MELTUMPs. This dual-marker algorithm promotes a shift from purely morphology-based evaluation toward a reproducible, molecularly informed classification, improving both diagnostic confidence and patient management.

## 1. Introduction

Cutaneous malignant melanoma is one of the most aggressive skin cancers, accounting for approximately 330,000 new diagnoses out of over 1.5 million cases of skin cancers reported worldwide in 2022 [1]. Historically, melanoma has been associated with a poor prognosis and high mortality; however, over the past two decades, significant improvements in early detection and the advent of targeted and immune therapies have markedly improved survival, particularly in Western countries [2,3].

Traditionally, melanocytic neoplasms have been classified within a rigid dichotomous framework, as either benign nevi or malignant melanomas [4]. However, this binary model fails to adequately encompass the full morphological spectrum of melanocytic proliferations. A subset of lesions presents with overlapping histopathological features that preclude definitive classification as benign or malignant. These diagnostically ambiguous lesions have been collectively termed Melanocytic Tumors of Uncertain Malignant Potential (MELTUMPs) [5]. MELTUMPs are characterized by melanocytic proliferations—often involving the dermis—that cannot be reliably categorized based on conventional histopathologic criteria. This heterogeneous group includes atypical Spitz tumors (ASTs), pigmented epithelioid melanocytoma, atypical deep penetrating nevi, atypical cellular blue nevi, cellular nodules within congenital nevi, and borderline melanoma variants such as minimal deviation melanoma [6,7].

Importantly, “MELTUMP” is a clinicopathological umbrella rather than a single biological entity. It encompasses lesions that arise through distinct molecular pathways, and this heterogeneity directly impacts the expected performance of 9p21 surrogates such as p16 and MTAP. For example, many blue-pathway tumors (blue nevus/blue melanocytoma and blue nevus-like melanoma) are driven by activating mutations in *GNAQ* or *GNA11* and may acquire additional alterations such as *BAP1*, *SF3B1* or *EIF1AX* during progression; in this setting, *CDKN2A* homozygous deletion is not necessarily the dominant event, and p16/MTAP may be less informative. Similarly, pigmented epithelioid melanocytoma and related tumors may be characterized by *PRKAR1A* inactivation and/or *PRKCA* fusions. Accordingly, p16/MTAP should be interpreted as pathway-dependent adjuncts: highly useful when 9p21-driven progression is plausible, but not a universal “solver” across the entire MELTUMP spectrum, where targeted molecular profiling may be more informative in selected categories (e.g., blue-pathway lesions/pigmented epithelioid melanocytoma (PEM)) [8,9,10].

These lesions share a key defining feature: architectural and/or cytologic atypia that exceeds the threshold for a benign nevus, yet lacks the unequivocal features required for a definitive diagnosis of melanoma.

Hence, accurate risk stratification of ambiguous melanocytic proliferations (including MELTUMPs and superficial atypical melanocytic proliferations of uncertain significance (SAMPUS)) remains crucial in order to guide management and follow-up, and to identify cases that warrant confirmatory molecular testing.

Current therapeutic approaches to the management of MELTUMPs are generally conservative. Sentinel lymph node biopsy is discouraged due to its high false-positive rate and limited prognostic value in this context [5,11]. Nevertheless, the diagnostic interpretation of MELTUMPs remains fraught with ambiguity and significant inter-observer variability, even among experienced dermatopathologists. Morphologically similar lesions may be variably interpreted as severely atypical nevi, MELTUMPs, or outright melanoma, which underscores the limitations of purely morphology-based assessment [12,13].

Given these challenges, diagnostic guidelines often err toward melanoma-oriented management strategies (such as wider excision margins) to avoid undertreatment. However, such an approach may contribute to substantial overtreatment, as most MELTUMPs ultimately follow an indolent clinical course. These issues highlight the pressing need for objective and reproducible ancillary tools to improve diagnostic accuracy and enable risk stratification within this gray zone of melanocytic pathology.

In this context, the evaluation of novel biomarkers has gained increasing importance. The present review will first outline the molecular foundations of the *CDKN2A*/*MTAP* axis on chromosome 9p21, followed by an appraisal of conventional and advanced immunohistochemical markers. We will then critically assess the diagnostic and potential prognostic role of combined p16 and Methylthioadenosine phosphorylase (MTAP) immunohistochemistry (IHC), exploring whether their integration can enhance diagnostic precision in ambiguous melanocytic lesions, particularly MELTUMPs.

## 2. Molecular Framework: The *CDKN2A/MTAP* Axis and the 9p21 Locus

Melanocytic tumorigenesis is deeply influenced by recurrent genetic alterations involving chromosome 9p21, a critical region that harbors key tumor suppressor genes, most notably *CDKN2A* and *MTAP*. Understanding the molecular architecture and biological interplay of these genes is essential for contextualizing their potential as diagnostic biomarkers in ambiguous melanocytic lesions, including MELTUMPs. These alterations represent early events in neoplastic progression and frame much of the current interest in immunohistochemical and molecular surrogates for risk stratification.

*CDKN2A/p16* is located on chromosome 9p21.3 and represents one of the most frequently altered tumor suppressor loci in human malignancies, such as melanoma, glioblastoma, pancreatic carcinoma, and familial melanoma syndromes. Uniquely, *CDKN2A* encodes two distinct proteins through alternative splicing and differential promoter usage: (1) p16^INK4a^, a cyclin-dependent kinase inhibitor responsible for regulating the G1 to S phase transition of the cell cycle by inhibiting CDK4 and CDK6, and (2) p14^ARF^ (p19^ARF^ in mice), which stabilizes the *p53* pathway through *MDM2* inhibition, promoting apoptosis and senescence. The loss of p16 function constitutes a pivotal event in melanoma pathogenesis. Mechanisms of inactivation include homozygous deletion, intragenic mutations, and epigenetic silencing via promoter hypermethylation. Functionally, p16 interferes with uncontrolled cell proliferation by maintaining the retinoblastoma (Rb) pathway in a hypophosphorylated, growth-suppressive state. In melanocytic pathology, the diffuse nuclear and cytoplasmic expression of p16 observed in benign nevi reflects a state of oncogene-induced senescence, acting as a protective barrier against malignant transformation [14]. Conversely, complete loss of p16 is strongly associated with homozygous *CDKN2A* deletion, a hallmark of melanoma progression. Nonetheless, not all melanomas exhibit p16 loss. This is because of their biological heterogeneity and the involvement of alternative oncogenic pathways.

MTAP is a ubiquitously expressed metabolic enzyme involved in the methionine and adenine salvage pathways, where it catalyzes the conversion of 5′-methylthioadenosine (MTA) into adenine and 5-methylthioribose-1-phosphate. Beyond its metabolic role, *MTAP* is increasingly recognized as a tumor suppressor gene [15,16,17]. Crucially, *MTAP* lies in close genomic proximity to *CDKN2A* on chromosome 9p21, approximately 100 kilobases telomeric. Due to this adjacency, large-scale homozygous deletions targeting *CDKN2A* often extend into *MTAP*, resulting in co-deletion. This biological linkage provides the rationale for investigating *MTAP* alongside *p16* as a combined biomarker system. In various malignancies, including mesothelioma, glioma, and melanoma, loss of *MTAP* expression correlates with advanced disease and increasingly aggressive clinical behavior [18,19,20]. *MTAP* deletion leads to intracellular accumulation of MTA, which in turn inhibits key methyltransferases and creates specific metabolic vulnerabilities. This lays the foundation for emerging synthetic lethality strategies that target protein arginine methyltransferase 5 (PRMT5), a downstream effector of MTAP. Loss of MTAP expression may thus both inform and enhance the therapeutic use of PRMT5 inhibitors [21].

Alterations of chromosome 9p21 are recognized as early events in melanocytic neoplasms. Several studies have demonstrated that loss of heterozygosity (LOH) and homozygous deletions at this locus occur not only in melanomas but also in dysplastic nevi, suggesting a role in the initial phases of tumorigenesis. In 1998, Won-Sang Park et al. investigated the frequency of deletions at the *p16* (in chromosome 9p21) and *p53* (in chromosome 17p13) tumor suppressor gene loci in premalignant and benign melanocytic lesions. Using microsatellite marker analysis, the researchers examined 9 dysplastic nevi and 13 benign intradermal nevi for LOH. A high frequency of LOH at one or more loci for *p16* was detected in 78% (7/9) of dysplastic nevi. In contrast, no LOH for *p16* was found in any of the benign intradermal nevi. LOH for p53 was also present in 43% (3/7) of informative dysplastic nevi, but importantly, all cases with *p53* deletion also harbored a *p16* deletion. These findings suggested that the deletion of the *p16* gene is a frequent and early genetic event in the development of dysplastic nevi, potentially preceding the loss of *p53* [22]. A few years later, other researchers investigated whether alterations at the p16/9p21 locus are early events in melanoma progression. Specifically, TP Tran et al. analyzed 44 cutaneous melanomas, 14 dysplastic nevi, and 6 benign nevi using LOH and homozygous deletion analysis. They found that LOH at 9p21 was highly prevalent in precursor lesions, occurring in 64% (9/14) of dysplastic nevi and 50% (3/6) of benign nevi, compared to 40% (17/44) of established melanomas. Furthermore, homozygous deletion of *p16* was identified in 29% (4/14) of dysplastic nevi but was completely absent in the benign nevi cohort. The authors concluded that LOH and homozygous deletions affecting the *p16* locus are early events in tumorigenesis, potentially preceding the development of cytological atypia. Data from individuals with multiple lesions suggested a progressive but heterogeneous pattern of genetic alterations, indicating that the 9p21 region may be affected at various stages during melanoma development [23].

Other studies have also suggested that these alterations may represent a critical early event in the molecular pathway of melanoma tumorigenesis. Specifically, 9p21 deletions are thought to contribute to melanocytic transformation and tumor initiation, while rearrangements at the *CDKN2A* locus and *p16* inactivation may play a key role in tumor progression [24]. To prove this, Zeng et al. [25] aimed to determine the frequency of *CDKN2A* coding region mutations in the atypical nevi of patients with sporadic melanoma. Using DNA isolated by laser-captured microdissection from the atypical nevi of 10 melanoma patients and their spouses as matched controls, researchers sequenced exons 1 and 2 of the *CDKN2A* gene. Interestingly, no point mutations in the *CDKN2A* coding region were identified in any of the melanocytic nevi analyzed. Therefore, *CDKN2A* point mutations could be considered a rare event in these precursor lesions. This finding may support a hypothesis where atypical nevi and melanoma represent separate, pleiotropic outcomes stemming from a common stimulus (e.g., UV-induced damage), rather than a direct linear progression.

Notably, another study uncovered the molecular mechanism linking the loss of the *CDKN2A* tumor suppressor to melanoma invasion and metastasis [26]. Using a CRISPR-Cas9-engineered model of melanoma initiation, the authors identified a direct signaling pathway where loss of *CDKN2A* leads to the activation of E2F1, which upregulates the lineage-restricted transcription factor BRN2. This finding was validated in a cohort of human melanocytic tumors, where *CDKN2A* loss coincided with the onset of invasion and increased BRN2 expression. Functionally, loss of the CDKN2A protein product, p16^INK4a^, promoted metastatic dissemination of human melanoma cells in a mouse model. This metastatic phenotype was rescued by the inhibition of BRN2. The authors, therefore, concluded that *CDKN2A* suppresses melanoma invasion by preventing the E2F1-mediated upregulation of BRN2, thereby identifying a key axis in melanoma progression. Additional studies confirmed that LOH can be identified even in dysplastic and certain benign nevi, whereas homozygous deletion is highly specific for melanoma or high-risk melanocytomas [22,26,27]. The co-deletion of *CDKN2A* and *MTAP* has been further associated with progression and increased metastatic potential. In diagnostic practice, assessment of 9p21 status through fluorescence in situ hybridization (FISH) or next-generation sequencing (NGS) can identify *CDKN2A* deletions and serve as a molecular anchor in ambiguous cases. Despite this, limitations related to accessibility, cost, and turnaround times have fueled interest in immunohistochemical surrogates—particularly *p16* and *MTAP*—as practical tools in routine pathology.

**Figure 1:**
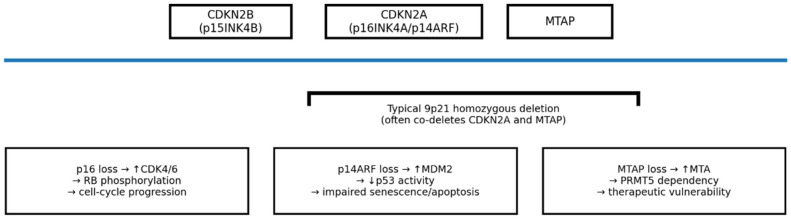
Schematic representation of the 9p21 locus (*CDKN2B–CDKN2A–MTAP*) and functional consequences relevant to melanoma progression and biomarker interpretation. Typical 9p21 homozygous deletions frequently co-delete *CDKN2A* and *MTAP*, linking concordant p16 and MTAP loss to a high likelihood of biallelic *CDKN2A* deletion. Functionally, *p16* (*CDKN2A*) loss releases *CDK4/6* activity, increasing *RB* phosphorylation and promoting G1–S cell-cycle progression, while loss of *p14^ARF^* impairs the *ARF–MDM2–p53* axis with reduced *p53*-mediated senescence/apoptosis. In parallel, MTAP loss leads to intracellular accumulation of 5′-methylthioadenosine (MTA), which can induce dependence on the *PRMT5/MAT2A* pathway and may represent a therapeutic vulnerability.

In particular we have schematically summarized the 9p21 locus and highlighted a key interpretive concept: the diagnostic endpoint with the highest specificity in ambiguous melanocytic lesions is *CDKN2A* homozygous deletion, which frequently co-deletes *MTAP* (Figure 1). Immunohistochemistry provides a practical surrogate readout (p16 and MTAP), but molecular methods remain required for definitive copy-number assessment. *CDKN2A* FISH directly identifies biallelic loss, while NGS-based panels with copy-number calling can capture both mutations and monoallelic/biallelic deletions (and may be complemented by methylation-based classifiers in selected settings). Importantly, heterozygous loss, point mutations, technical variables, and intratumoral heterogeneity can yield partial or heterogeneous p16 patterns with retained MTAP; therefore, discordant or equivocal IHC profiles (particularly in small biopsies or heavily pigmented lesions) should prompt reflex molecular testing rather than categorical interpretation. A critical interpretive nuance involves distinguishing between monoallelic (heterozygous) loss and biallelic (homozygous) deletion. While heterozygous alterations or point mutations often yield heterogeneous or ‘mosaic’ p16 patterns with retained MTAP expression, only the concordant and complete loss of both markers serves as a robust surrogate for the biallelic *CDKN2A*/*MTAP* deletion that characterizes malignant progression.

## 3. Conventional Immunohistochemical Markers in Ambiguous Melanocytic Lesions

IHC remains a cornerstone in the evaluation of melanocytic lesions, particularly when morphology alone is insufficient to determine malignancy. In routine practice, a panel of conventional melanocytic markers is employed to support lineage identification and assess proliferative activity. However, although these markers are sensitive for melanocytic differentiation, their role in distinguishing benign from malignant lesions, especially within the MELTUMP spectrum, is limited.

Among the classical melanocytic lineage markers, S100 protein represents the most sensitive indicator of melanocytic cells and is consistently expressed in both benign nevi and malignant melanomas. Despite its high sensitivity, S100 protein lacks specificity, and this restricts its value in diagnostic discrimination [13]. HMB-45, which targets gp100 (a premelanosomal glycoprotein), is more specific for melanocytic differentiation and frequently shows a maturation gradient in benign nevi, with diminishing expression in deeper lesional cells. In contrast, melanomas, particularly nodular and epithelioid variants, may demonstrate diffuse or aberrant expression patterns [13,20]. Melan-A (MART-1) provides both sensitivity and specificity for melanocytic lineage and is routinely used alongside HMB-45; however, it does not reliably distinguish between benign and malignant proliferations, as expression can be retained in atypical and borderline lesions [20]. More recently, SOX10, a nuclear transcription factor, has emerged as a superior marker for melanocytic and neural crest-derived tumors. SOX10 is particularly valuable in desmoplastic melanoma and spindle cell variants, where other markers such as HMB-45 and Melan-A may be negative. Nonetheless, despite its diagnostic utility, SOX10 remains a lineage marker rather than a discriminator of malignancy.

Proliferation and cell cycle markers also contribute to diagnostic assessment. Ki-67 (MIB-1), a well-established proliferation index marker, is useful in differentiating benign proliferations, which typically exhibit indices below 5%, from malignant lesions, which exhibit indices that often exceed 10–15%. Elevated Ki-67 values correlate with aggressive biological behavior, yet significant overlap persists in borderline entities such as ASTs [27]. In addition, immune checkpoint markers such as PD-L1 are increasingly being assessed to characterize the tumor microenvironment and predict responsiveness to immunotherapy. While these immune checkpoint markers are not diagnostic, their expression may carry prognostic or therapeutic relevance in advanced melanoma [28,29].

Despite the pivotal role conventional IHC plays in confirming melanocytic lineage, its capacity to aid in risk stratification or definitively separate benign from malignant lesions remains limited. This limitation is particularly evident in MELTUMPs, where overlapping histomorphological and immunophenotypic features persist even after extensive immunohistochemical evaluation. Several challenges contribute to this diagnostic ambiguity, including morphologic mimicry among atypical nevi, Spitz tumors, and melanoma, intratumoral heterogeneity with the coexistence of benign and malignant features, and substantial inter-observer variability, even among subspecialty-trained dermatopathologists. Furthermore, the absence of standardized biomarkers for borderline entities further complicates diagnostic consensus, even if we can consider a panel of immunohistochemical biomarkers employed in the diagnostic workup of melanoma and related lesions (Table 1). Given these limitations, increasing attention has shifted toward molecularly anchored immunohistochemical surrogates such as *p16* and *MTAP*, which reflect underlying genomic alterations rather than solely phenotypic expression.

## 4. p16 IHC: Diagnostic Significance and Limitations

Among cell cycle regulators, *p16* has attracted particular interest in melanocytic pathology due to its potential role in distinguishing benign nevi—including Spitz nevi, which generally retain strong and diffuse nuclear and cytoplasmic p16 immunohistochemical expression—from melanomas, where loss or marked reduction is frequently observed. This phenomenon is often interpreted as a manifestation of oncogene-induced senescence, a protective cellular mechanism that restricts proliferation in response to oncogenic stress, preventing malignant transformation. In contrast, substantial loss or complete absence of *p16* expression is frequently encountered in malignant melanoma, where it represents a surrogate marker of *CDKN2A* inactivation, typically due to homozygous deletion, mutation, or promoter hypermethylation. This biological dichotomy has positioned *p16* as a potential ancillary marker in the evaluation of diagnostically ambiguous melanocytic lesions.

Several studies have explored the diagnostic significance of *p16*, particularly in differentiating benign lesions from melanomas within the spectrum of dermal-based proliferations. For example, Oaxaca et al. [30] evaluated the diagnostic role of *p16* IHC in a heterogeneous cohort of 104 dermal-based melanocytic neoplasms, identifying two main staining patterns: a single homogeneous profile and a heterogeneous profile. The most critical observation was that total loss of *p16* expression, whether diffuse or regional, occurred exclusively in melanomas. This criterion showed high specificity for malignancy, identifying 61% of melanomas (14 out of 23) while being entirely absent in all benign nevi. The authors concluded that complete *p16* loss is a strong indicator of melanoma and that the presence of a heterogeneous expression pattern warrants careful pathological assessment, reinforcing the value of *p16* IHC as an ancillary tool in challenging melanocytic lesions. However, despite the promise of *p16* as a diagnostic marker, its use is marred by variability in expression among melanomas and overlap in staining patterns with benign lesions. Reed et al. [31] investigated the temporal dynamics of *p16* loss during melanoma progression by analyzing its immunohistochemical expression in 103 melanocytic lesions. They found that *p16* expression was retained in all early-stage lesions, including melanoma in situ and most primary invasive melanomas. Significant loss of expression (partial or complete) emerged predominantly in advanced disease, being observed in 52% of primary invasive tumors and 72% of metastatic lesions. These observations suggest that *p16* loss is not an initiating event in melanoma tumorigenesis, but that it may instead represent a later development associated with tumor progression and acquisition of invasive potential.

Scurr et al. [32] challenged the interpretation of *p16* loss as a late event. Analyzing 20 benign and dysplastic nevi, they observed that all lesions exhibited a heterogeneous pattern of *p16* immunohistochemical expression, which suggests that *p16* dysregulation may occur early in lesion development and possibly contributes to initial melanocytic proliferation rather than exclusively signaling malignant transformation. A systematic review and meta-analysis by Chinchanikar et al. [33] further highlighted the limitations of *p16* as a standalone diagnostic biomarker. Analyzing 26 studies comprising 979 melanomas and 974 nevi, the authors reported that the diagnostic performance of *p16* loss is limited, with a pooled sensitivity of 0.55 (95% CI: 0.38–0.70) and specificity of 0.85 (95% CI: 0.70–0.94). These values fall below the threshold for a reliable diagnostic assay, underscoring a fundamental trade-off: while complete loss of *p16* can be highly specific for malignancy (particularly in distinguishing melanoma from common nevi, Spitz nevi, or deep penetrating lesions), retained expression cannot definitively exclude it. This intrinsic trade-off between sensitivity and specificity limits the use of *p16* when applied in isolation.

Nowadays, the clinical application of *p16* is further complicated by interpretive nuances, including variability in staining patterns (diffuse, focal, mosaic), the need to distinguish nuclear from cytoplasmic staining, and the absence of universally accepted scoring criteria. While some authors advocate strict nuclear assessment, others accept combined nuclear and cytoplasmic patterns, and this contributes to inter-observer variability. Moreover, external factors such as fixation quality and antibody clone selection may influence staining results, introducing additional layers of technical complexity.

In the context of MELTUMPs, where diagnostic ambiguity is inherent, reliance on *p16* alone risks both overdiagnosis and underdiagnosis. Retained *p16* expression does not preclude malignancy, particularly in tumors driven by alternative pathways such as *BRAF* or *NRAS* mutations, while loss of expression may occur in borderline lesions lacking overt malignant behavior. These observations underscore the necessity of integrating *p16* evaluation with additional biomarkers and clinical-pathologic correlation [34]. For these reasons, recent research has shifted toward combining *p16* with complementary biomarkers such as PRAME or MTAP to enhance diagnostic performance through multimodal evaluation [35]. While *p16* remains a cornerstone of immunohistochemical investigation in melanocytic lesions, its role has evolved from a putative sentinel marker to a component within a broader diagnostic matrix.

## 5. MTAP IHC: Specificity and Clinical Implications

Recently, MTAP has emerged as a relevant biomarker in the context of melanocytic neoplasms due to its dual biological and diagnostic significance. Located on chromosome 9p21, in close genomic proximity to *CDKN2A*, the *MTAP* gene is frequently subject to co-deletion in tumors exhibiting homozygous loss of *p16*. While *p16* reflects alterations in cell cycle regulation, *MTAP* represents a metabolic tumor suppressor involved in the salvage pathway of adenine and methionine through the phosphorolysis of methylthioadenosine. The combined loss of *CDKN2A* and *MTAP* is a recurrent event in several malignancies, including mesothelioma, glioma, and melanoma, suggesting a broader biological linkage within the 9p21 locus [17,18,19].

Early experimental studies by Behrmann et al. [36] demonstrated a significant reduction in *MTAP* mRNA and protein levels in melanoma cell lines. Mechanistically, this downregulation could result from promoter hypermethylation or, in cases of large deletions, complete gene loss. Subsequent in vivo investigations corroborated these findings, revealing a progressive decrease in *MTAP* expression from benign nevi to metastatic melanoma [22,23]. This gradient of expression suggested that *MTAP* inactivation may contribute not only to tumor development but also to disease progression. In addition, Kvaskoff et al. [37] conducted a large-scale meta-analysis of 11 genome-wide association studies (GWAS) involving 52,506 individuals to identify genetic loci associated with nevus count—a major risk factor for cutaneous melanoma. The analysis confirmed previously established nevus-associated loci (*MTAP*, *PLA2G6*, *IRF4*) and uncovered novel associations with single-nucleotide polymorphisms (SNPs) in *KITLG* and a region on chromosome 9q32. To explore the genetic overlap between nevus count and melanoma risk, the researchers performed a bivariate meta-analysis by integrating the nevus data with a large melanoma GWAS (12,874 cases and 23,203 controls). This analysis revealed several additional shared susceptibility loci reaching genome-wide significance, including SNPs near *GPRC5A*, *CYP1B1*, *PPARGC1B*, *HDAC4*, *FAM208B*, *DOCK8*, and *SYNE2*. The findings support a model of substantial but incomplete genetic overlap between nevus formation and melanoma susceptibility. Interestingly, the authors proposed that most genes that influence nevus count also modulate melanoma risk—with *KITLG* representing a notable exception—whereas many known melanoma risk loci do not affect nevus propensity. The results implicate multiple biological pathways in nevogenesis and refine our understanding of the shared genetic architecture underlying nevi and melanoma.

To further explore the hypothesis that melanomas arise through distinct “nevus-associated” and “chronic sun exposure” pathways, the authors analyzed associations between nevus-related loci (*MTAP*, *PLA2G6*, *IRF4*) and melanoma risk in 1028 cases and 1469 controls, stratified by histological subtype and anatomical site. They found that the *MTAP* variant rs10757257 was significantly associated with overall melanoma risk (OR = 1.32, 95% CI = 1.14–1.53), specifically for superficial spreading and nodular melanoma subtypes (OR = 1.34), but not for lentigo maligna melanoma (*p* for homogeneity = 0.06), which is typically linked to chronic sun exposure. In contrast, the *IRF4* variant rs12203592 showed an age-dependent effect, conferring a significant protective association only among children and adolescents (*p* for homogeneity = 0.0008). Collectively, these findings provide genetic evidence supporting the “divergent pathways” model of melanoma development. The subtype-specific association of the *MTAP* locus identifies it as a plausible candidate for the nevus-associated pathway, while the age-dependent effect of *IRF4* underscores the heterogeneity of melanoma susceptibility and the complex interplay between genetic and environmental factors.

Additionally, the potential clinical relevance of *MTAP* expression in melanoma was further evaluated in studies assessing its association with clinicopathologic features and therapeutic response.

To identify potential correlations of *MTAP* expression with clinicopathologic variables and patient outcomes, Wild et al. [38] applied tissue microarrays in the analysis of MTAP protein expression in a large cohort of 315 benign and malignant melanocytic skin tumors. Cytoplasmic MTAP expression was detected in 227 of the 315 informative cases (72.1%) and significantly reduced in primary malignant melanomas and melanoma metastases compared with benign nevi (*p* < 0.001 for both). No significant difference in *MTAP* expression was observed between primary melanomas and their metastatic lesions. Within primary melanomas, loss of *MTAP* expression correlated with increased cellular proliferation, as indicated by a Ki67-labeling index ≥ 5% (*p* = 0.04). Furthermore, lymph node metastases displayed higher *MTAP* levels than skin metastases (*p* = 0.01), suggesting possible site-specific modulation of expression.

Although *MTAP* expression was not associated with overall prognosis across the full cohort, a significant predictive pattern emerged in the subset of patients who developed recurrent disease. Among the 26 patients with MTAP-positive melanomas and tumor recurrence, those who received adjuvant interferon therapy (*n* = 18) exhibited a significant survival benefit compared to those who did not receive interferon (*n* = 8) (*p* = 0.009). Importantly, no such therapeutic advantage was observed in patients with MTAP-negative tumors, suggesting that *MTAP* expression may predict sensitivity to interferon therapy. This hypothesis was further supported by a subsequent study by Meyer et al. [39], which confirmed that MTAP-positive melanomas derive significant survival benefit from adjuvant interferon, whereas MTAP-negative tumors show no therapeutic response.

Taken together, these findings indicate that *MTAP* loss is associated with malignant progression and increased proliferative activity in melanoma, while retained *MTAP* expression may predict responsiveness to interferon therapy. Although further validation in larger, prospective cohorts is warranted, *MTAP* status emerges as a promising predictive biomarker of interferon sensitivity, potentially expanding its clinical application beyond diagnostic stratification. From a purely diagnostic perspective, *MTAP* has gained attention due to its exceptionally high specificity when evaluated by IHC. Unlike *p16*, which may demonstrate patchy or mosaic expression, *MTAP* loss tends to follow a binary pattern of either complete cytoplasmic absence or retention in tumor cells, accompanied by intact staining in internal non-neoplastic controls such as stromal fibroblasts or inflammatory infiltrates. Multiple studies across tumor types have reported specificity values approaching or reaching 97–100% for homozygous 9p21 deletion when *MTAP* expression is absent [18,40,41,42,43,44,45]. This high specificity positions *MTAP* as a valuable confirmatory marker in cases where *p16* interpretation is equivocal.

Recent evidence has refined the diagnostic interpretation of *MTAP* IHC in melanocytic lesions. In a comprehensive study of 45 nevi and 70 melanomas with molecular correlation, He et al. [46] demonstrated that *MTAP* loss is a highly specific surrogate marker for *CDKN2A* homozygous deletion, though with limited sensitivity. In their series, *MTAP* loss was observed in 10% of melanomas and in none of the nevi, while *p16* loss occurred in 59% of melanomas. When correlated with molecular data, *MTAP* loss showed 100% specificity and a positive predictive value of 100% for detecting *CDKN2A* homozygous deletion, but only 41% sensitivity, confirming that *MTAP* immunostaining alone lacks optimal sensitivity for detecting all *CDKN2A*-inactivated melanomas. This suboptimal sensitivity reflects the molecular architecture of the 9p21 locus, where smaller deletions may selectively involve *CDKN2A* while sparing the adjacent *MTAP* gene, resulting in melanomas that are p16-negative but retain *MTAP* expression. Conversely, *MTAP* loss is entirely specific for larger deletions encompassing both *CDKN2A* and *MTAP*, which also correspond to homozygous *MTAP* locus deletion with 100% specificity and a positive predictive value of 100%. Therefore, while *MTAP* IHC represents an excellent confirmatory marker for homozygous *CDKN2A* deletion, its limited sensitivity prevents its use as a standalone screening tool. Combined assessment of *p16* and *MTAP* expression provides complementary diagnostic information, with *p16* serving as a more sensitive marker and *MTAP* providing absolute specificity for 9p21 homozygous loss. Importantly, *MTAP* loss may also identify tumors potentially susceptible to PRMT5 or related methyltransferase inhibitors, given the functional link between *MTAP* deletion and PRMT5 pathway dependency.

Accordingly, a diagnostic strategy that relies exclusively on *MTAP* may yield false-negative interpretations in such contexts. The distinction between biological and diagnostic relevance must therefore be carefully maintained. While *p16* offers higher sensitivity but limited specificity, *MTAP* provides absolute specificity for homozygous *CDKN2A* deletion, albeit with lower sensitivity. These complementary characteristics support the rationale that simultaneous assessment of both markers may achieve greater diagnostic accuracy, particularly in the evaluation of MELTUMPs and other borderline melanocytic proliferations. In particular, the interpretation of MTAP IHC also requires awareness of potential pitfalls, including deceptive focal staining in inflammatory or stromal cells and misinterpretation of weak, granular cytoplasmic signals as retained expression. Despite these nuances, interobserver reproducibility for *MTAP* appears superior to that of *p16*, owing to its clearer dichotomous (retained vs. lost) staining pattern.

Beyond diagnostics, the *p16*/*MTAP* axis may have emerging therapeutic relevance, although mostly within clinical trial settings. Loss of *p16* reflects deregulation of the *CDK4/6–RB* checkpoint and provides a biological rationale for exploring *CDK4/6* inhibitors in molecularly selected melanoma subsets. Similarly, *MTAP* loss creates a metabolic context (MTA accumulation) that can confer *PRMT5*-pathway dependency and has motivated the development of *PRMT5/MAT2A*-axis strategies in *MTAP*-deleted solid tumors. At present, these considerations should be interpreted as potential translational implications rather than routine treatment-selection criteria for borderline lesions [47,48].

## 6. Combined p16/MTAP Assessment and Integrative Diagnostic Strategies

*MTAP* IHC occupies a distinct and complementary role in melanocytic diagnostics, situated at the intersection of molecular biology and applied pathology. The greatest value lies in its integration with *p16* within a combined immunohistochemical framework, where its high specificity can counterbalance the greater sensitivity of *p16*. In this dual-marker paradigm, *MTAP* not only refines the classification of diagnostically ambiguous melanocytic neoplasms but may also hold therapeutic implications, identifying tumors potentially susceptible to PRMT5 and related methyltransferase inhibitors in the context of 9p21 codeletion. Therefore, the diagnostic value of *p16* and *MTAP* reaches its greatest potential when these biomarkers are interpreted in combination as part of an integrated immunohistochemical strategy. Clinical studies on other malignancies have already demonstrated the diagnostic power of this combined strategy. In mesothelioma and glioma, for instance, concordant loss of *p16* and *MTAP* has been shown to correlate strongly with *CDKN2A/B* deletion, outperforming either marker alone [18,43,44,45]. Emerging data in melanoma suggest similar potential. Although the body of literature remains limited, retrospective studies have consistently shown that combined loss of *p16* and *MTAP* expression is strongly associated with lesions ultimately classified as melanoma or high-risk melanocytomas [46]. In contrast, discordant immunophenotypic profiles such as p16-negative but MTAP-retained expression are more frequently observed in borderline or intermediate melanocytic proliferations lacking unequivocal malignant potential, underscoring the molecular heterogeneity of 9p21 alterations [49]. In this context, R. Vergara et al. [41] conducted a retrospective study to evaluate the diagnostic utility of integrating *p16* IHC with 9p21 FISH analysis in a cohort of 206 diagnostically challenging melanocytic tumors referred for expert consultation. Their findings demonstrated that complete loss of *p16* expression was a highly sensitive marker for malignancy, identifying 90% of malignant cases, whereas only 11% of benign lesions exhibited *p16* loss. Conversely, homozygous 9p21 deletion detected by FISH showed high specificity, being present in only 5% of benign lesions, although its sensitivity was limited at 42%. Heterozygous deletions, however, were found to be diagnostically irrelevant. The authors concluded that the combined assessment of *p16* and 9p21 status represents a practical and effective strategy to enhance diagnostic accuracy in ambiguous melanocytic tumors while recommending additional molecular investigations in cases with discordant or inconclusive results to explore alternative mechanisms of *p16* inactivation or other genomic alterations.

While MELTUMP is often used as a practical diagnostic label for lesions with uncertain malignant potential, it aggregates biologically distinct entities. As a result, the utility of p16/MTAP is not uniform and should be calibrated to the expected pathway of progression. In lesions in which 9p21 alteration is a recognized progression-associated event (e.g., a subset of spitzoid tumors and deep penetrating neoplasms with worrisome features), concordant *p16* and *MTAP* loss can provide strong supportive evidence for biologically aggressive behavior and justify reflex confirmation of *CDKN2A* homozygous deletion by FISH or NGS-CNV, alongside clinical-pathologic correlation. Conversely, in blue-pathway lesions (*GNAQ*/*GNA11*-driven) and in pigmented epithelioid melanocytoma, progression is frequently shaped by alternative drivers (e.g., *BAP1*/*SF3B1*/*EIF1AX* or *PRKAR1A*/*PRKCA*), and 9p21 surrogates may be less discriminative; in such contexts, early targeted molecular profiling may be preferable when a definitive risk assessment is needed. Accordingly, p16/MTAP should be framed as tools to reduce uncertainty and guide reflex testing, not as stand-alone markers that can resolve every MELTUMP subtype into fixed categories [8,9,10].

Further insight into the role of the 9p21 region in melanomagenesis was provided by G. Palmieri et al. [50], who investigated genetic alterations across this chromosomal locus in sporadic malignant melanoma to differentiate events involved in tumor initiation versus progression. By analyzing 66 primary melanomas and 58 matched metastases for LOH and microsatellite instability (MSI) using nine polymorphic markers, the study revealed that LOH was a frequent and early event, detected in 41% of primary tumors and 47% of metastases. In contrast, MSI was more prevalent in metastatic lesions (38%) compared to primary tumors (17%), suggesting its association with disease advancement. While the *CDKN2A* locus was confirmed as the primary target of allelic loss, a second recurrent deletion was detected at the D9S171 marker, suggesting the existence of an additional melanoma susceptibility gene within the 9p21 region. Importantly, no significant associations were found between these genetic alterations and clinicopathological features.

Taken together, these findings support the implementation of a tiered diagnostic algorithm in challenging melanocytic lesions, particularly within the MELTUMP spectrum. Lesions retaining both *p16* and *MTAP* expression may be managed more conservatively, emphasizing clinicopathological correlation before resorting to ancillary testing. Conversely, concordant loss of both markers should prompt strong consideration for reclassification toward melanoma and may justify reflex molecular studies such as FISH or NGS to confirm genomic deletion. Finally, discordant cases (especially those with isolated *p16* loss and preserved *MTAP* expression) highlight the biological complexity of 9p21 alterations and underscore the need to incorporate additional adjunctive markers such as PRAME or expanded genetic panels to refine diagnostic interpretation and risk stratification.

PRAME, a cancer-testis antigen overexpressed in melanoma, has emerged as a useful adjunct marker in differentiating benign from malignant melanocytic lesions. When interpreted alongside *p16* and *MTAP*, PRAME may further enhance diagnostic precision, particularly in deep penetrating neoplasms or spitzoid lesions with ambiguous features [51]. Nevertheless, caution is warranted, as PRAME expression can occasionally be detected in atypical nevi, and its absence does not exclude malignancy.

The adoption of a p16/MTAP-based diagnostic framework has the potential to shift the current paradigm away from morphology-only assessment toward an increasingly molecularly informed practice. However, this transition must be grounded in carefully validated evidence. Standardized scoring criteria, appropriate internal controls, and awareness of interpretive pitfalls remain essential to ensure reproducibility. While not all laboratories have access to advanced molecular platforms such as FISH or NGS, p16 and MTAP IHC offer a practical, cost-effective alternative that may be readily implemented in routine diagnostic settings. Despite its promise, the combined use of *p16* and *MTAP* should not be perceived as a definitive solution. These markers provide valuable guidance but must be contextualized within a broader clinicopathological framework that includes patient age, lesion location, morphologic architecture, cytologic features, and, when necessary, molecular correlation. The true strength of the combined approach lies not in absolute categorization but in its ability to reduce diagnostic uncertainty, narrow differential diagnoses, and identify lesions warranting closer scrutiny or more aggressive management. As research progresses, it is conceivable that *p16* and *MTAP* will occupy a central position in future diagnostic algorithms for MELTUMPs, potentially integrated with genomic classifiers and artificial intelligence-based pattern recognition. Their ultimate role may not be in replacing morphology but in complementing it, enabling a shift from subjective diagnosis to data-supported classification.

**Figure 2:**
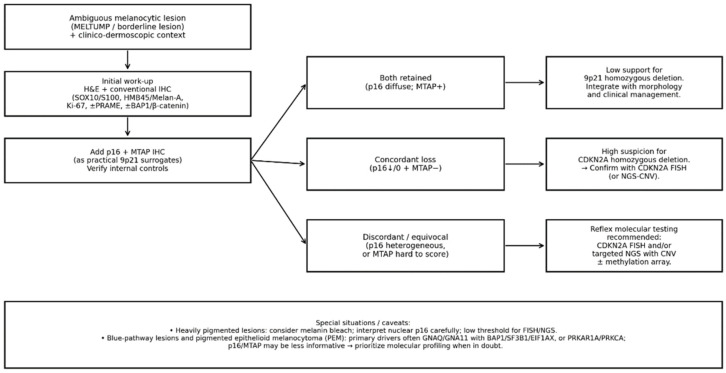
Proposed stepwise diagnostic workflow integrating p16 and MTAP immunohistochemistry (IHC) with morphology and reflex molecular testing in ambiguous melanocytic lesions (including the MELTUMP spectrum). After establishing the morphological differential diagnosis on H&E (hematoxylin and eosin) sections and integrating the clinical/dermoscopic context, a conventional immunopanel should be performed (e.g., SOX10/S100; HMB45/Melan-A; Ki-67; ±PRAME; ±BAP1 and/or β-catenin depending on the differential). When 9p21-driven progression is plausible and a molecular “anchor” is needed, p16 (CDKN2A) and MTAP should be added as practical surrogates of 9p21 status, with interpretation guided by internal controls and pattern-based rules. Both retained (diffuse p16 with MTAP positivity) provide low support for 9p21 homozygous deletion and favor management by integrated clinicopathological risk assessment. Concordant loss (complete or near-complete loss of p16 in the tumor, typically >90% of cells or clear clonal loss, together with MTAP negativity) raises high suspicion for biallelic *CDKN2A* deletion, prompting confirmation by *CDKN2A* FISH and/or NGS with CNV (copy-number variation) calling. Discordant/equivocal results (heterogeneous p16, difficult-to-score MTAP, or technical uncertainty) should not be over-interpreted and instead trigger reflex molecular testing (FISH/NGS ± methylation array), with attention to sampling limitations. Technical caveats (heavy pigmentation, fixation issues, small biopsies) warrant a lower threshold for molecular confirmation.

To facilitate implementation in routine practice, we propose a stepwise workflow (Figure 2) in which p16/MTAP are ordered after morphology and a conventional immunopanel, and interpreted in three actionable patterns: (i) both retained (low support for 9p21 homozygous deletion), (ii) concordant loss (high suspicion; confirm with *CDKN2A* FISH or NGS-CNV (copy-number variation)), and (iii) discordant or equivocal profiles (reflex molecular testing recommended). Special situations include heavily pigmented lesions (where cytoplasmic MTAP may be difficult to score and nuclear p16 should be interpreted cautiously) and blue-pathway/PEM tumors (where primary drivers may not be 9p21-driven).

## 7. Standardization of p16 and MTAP Immunohistochemical Assessment in Melanocytic Lesions: Toward Harmonized Scoring and Diagnostic Reproducibility

The diagnostic value of *p16* and *MTAP* IHC depends as much on interpretive consistency as on biological specificity. Unlike lineage-confirming markers such as S100 or Melan-A, these two biomarkers require careful assessment of staining distribution, intensity, and internal control integrity. A persistent obstacle to their routine diagnostic adoption is the lack of harmonized scoring systems, which has led to considerable variability across studies and laboratories. Early investigations, such as that by Reed et al. (1995) [31], first established the diagnostic relevance of *p16* loss in melanoma through qualitative assessment of nuclear and cytoplasmic staining. While this approach yielded high specificity for malignancy, its limited sensitivity highlighted the pitfalls of purely binary evaluation. Subsequent work by Al Dhaybi et al. (2011) [52] in pediatric spitzoid lesions adopted a simplified positive/negative model, demonstrating near-perfect concordance between *p16* loss and malignant transformation, although its applicability remained constrained by small cohort size and lack of quantitative validation. Building on these observations, Yazdan et al. (2014) [49] compared the immunohistochemical patterns of *p16* in ASTs with heterozygous versus homozygous 9p21 deletions. Using a semi-quantitative 0–3 scoring system based on nuclear staining extent, they found that *p16* expression was retained in approximately two-thirds of heterozygous cases but completely lost in all tumors with homozygous deletion. This study provided pivotal evidence that complete *p16* loss correlates with biallelic 9p21 deletion, whereas partial or patchy loss may reflect heterozygous alterations or non-neoplastic variability, emphasizing the need for integrated molecular correlation in interpreting *p16* IHC. More nuanced frameworks were later introduced by Harms et al. (2016) [53], who combined both qualitative and H-score–based systems to capture partial or mosaic expression, revealing that intermediate or heterogeneous *p16* loss may occur in ASTs. The H-score, a semi-quantitative index calculated as the product of staining intensity (0–3) and the percentage of positive cells (0–100), provides a composite value (range 0–300) reflecting both extent and strength of immunoreactivity. These findings reinforced the notion that *p16* interpretation cannot rely solely on percentage cut-offs but that it could integrate staining intensity and distribution.

Regarding *MTAP*, the interpretation of staining presents distinct challenges due to its cytoplasmic localization. Evaluation requires comparison with strong cytoplasmic staining in non-neoplastic internal controls, including fibroblasts, endothelial cells, and inflammatory cells. Early observations by Behrmann et al. (2003) [36] suggested a link between cytoplasmic *MTAP* loss and melanoma progression, paving the way for its recognition as a potential marker of biological aggressiveness. More recently, He et al. (2025) [46] provided the most comprehensive molecularly correlated analysis to date, demonstrating that concurrent loss of *p16* and *MTAP* strongly aligns with homozygous 9p21 deletion, while discordant or mosaic patterns warrant cautious interpretation. Technical and pre-analytical variables further complicate interpretation. Fixation time, antigen retrieval, antibody clone, and staining platform can markedly influence staining quality. Over-fixation in formalin may reduce nuclear *p16* intensity, mimicking partial loss, while background inflammatory staining or macrophage uptake can obscure *MTAP* expression. The absence of positive internal controls renders slides non-interpretable rather than negative. Moreover, intratumoral heterogeneity—particularly in melanocytic lesions with mixed cellular subclones—can yield sampling bias, where retained clones are overrepresented in small biopsies, resulting in false-negative assessments. To address these interpretive challenges, recent studies have emphasized the need for standardized, algorithmic frameworks that integrate staining patterns, internal controls, and molecular correlates [46,47]. Rather than relying on any single marker, these models promote a tiered interpretive approach in which immunohistochemical results are weighed alongside morphologic and clinical data. However, the reproducibility of such combined strategies across laboratories remains to be systematically validated. Progress toward consensus scoring systems—particularly regarding thresholds, internal control criteria, and interpretive hierarchies—will be essential for the reliable incorporation of *p16* and *MTAP* IHC into diagnostic workflows. Finally, these markers illustrate how dermatopathology is evolving from a morphology-based to a molecularly informed discipline, in which biologically grounded markers are interpreted through reproducible, standardized, and clinically meaningful frameworks. Therefore, it has been suggested that representative scoring systems with interpretive cut-offs for *p16* and *MTAP* IHC across different studies illustrate the heterogeneity of current practice and the need for harmonized, reproducible interpretive frameworks (Table 2).

In cases of heavy melanin pigmentation, which may obscure cytoplasmic MTAP signals or interfere with nuclear p16 evaluation, the use of technical adjuncts is strongly recommended. Specifically, melanin bleaching protocols (e.g., using dilute hydrogen peroxide) or the employment of red chromogens (such as 3-Amino-9-ethylcarbazole (AEC)) can significantly improve diagnostic interpretability by distinguishing the brown-black endogenous pigment from the immunohistochemical reaction product.

## 8. Conclusions and Future Perspectives

The diagnostic evaluation of MELTUMPs represents one of the most demanding challenges in dermatopathology, where conventional morphology and standard IHC often reach the limits of interpretive certainty. Within this complex landscape, the combined use of *p16* and *MTAP* IHC has emerged as a promising strategy to refine diagnostic judgment, providing a biologically grounded surrogate for genomic alterations involving the 9p21 locus. Individually, each marker offers distinct strengths and limitations. Long regarded as a functional indicator of *CDKN2A* inactivation, *p16* demonstrates considerable sensitivity but insufficient specificity, particularly in borderline lesions where mosaic staining patterns confound interpretation. In contrast, *MTAP* offers exceptional specificity, reliably identifying cases harboring homozygous 9p21 deletion; however, its lower sensitivity limits its use as a solitary screening tool. When interpreted together, these biomarkers provide complementary insight: concordant loss of *p16* and *MTAP* strongly supports the presence of underlying genomic deletion and, in the appropriate clinicopathological context, reinforces a diagnosis of melanoma rather than uncertain or intermediate lesions. Despite this synergistic potential, the integration of *p16* and *MTAP* into routine practice necessitates caution. Interpretation remains susceptible to technical variability, intratumoral heterogeneity, and the absence of standardized scoring criteria. Equivocal staining patterns, absence of internal controls, and discordant profiles should prompt careful reevaluation rather than immediate diagnostic escalation. These markers are not a substitute for morphology but rather an extension of it, and their true value lies in reducing diagnostic ambiguity rather than providing absolute categorization.

Looking ahead, the future of MELTUMP classification will likely depend on the integration of molecularly anchored biomarkers such as *p16* and *MTAP* within broader diagnostic frameworks that incorporate ancillary tools, including PRAME IHC, FISH, SNP arrays, and NGS. The development of validated diagnostic algorithms, supported by multicenter studies and consensus guidelines, will be essential for establishing reproducible criteria capable of transcending individual expertise and institutional variability. In this pursuit, *p16* and *MTAP* stand not as final solutions but as foundational components of a more precise, biologically informed diagnostic paradigm. Ultimately, the incorporation of p16/MTAP assessment reflects a broader transition in dermatopathology: from reliance on morphologic impression to the adoption of integrated, molecularly guided decision-making. Overall, the combined interpretation of p16 and MTAP is best viewed as a reproducible, pathway-informed adjunct that can reduce diagnostic uncertainty and support decisions on when to pursue confirmatory molecular testing (*CDKN2A* FISH/NGS-CNV), rather than as a tool that redefines MELTUMP entities or substitutes for driver-based classification. In the near term, its most practical contribution may be to identify cases with a high likelihood of 9p21 homozygous deletion, to flag discordant/equivocal profiles that merit reflex testing, and to standardize communication of risk in multidisciplinary settings. Future prospective studies stratified by MELTUMP subtype and molecular pathway will be required to validate outcome associations and to determine how best to integrate p16/MTAP with genomic and methylation-based classifiers, mirroring the multi-modal risk-stratification approaches already adopted in other tumor settings [54,55,56].

## Figures and Tables

**Table 1:** The key immunohistochemical markers used in the diagnosis and prognosis of melanocytic lesions.

Category	Marker	Type	Diagnostic Role	Prognostic/Predictive Role
Immunohistochemical markers for melanocytic lineage (not specific)	S100 Protein	Protein	Very sensitive marker for melanocytic differentiation	Not prognostic
	HMB-45	Protein	Specific for melanocytic differentiation; useful in conjunction with other markers	Limited prognostic role
	Melan-A (MART-1)	Protein	Sensitive and specific for melanocytes; supports diagnosis	Not prognostic
	SOX10	Transcription factor	Sensitive nuclear marker for melanocytic lesions	Not prognostic
Proliferation marker	Ki-67 (MIB-1)	Proliferation index	Helps distinguish benign from malignant lesions	Higher index correlates with aggressive behavior
Immune checkpoint marker	PD-L1	Immune checkpoint ligand	May support differential diagnosis in the tumor microenvironment	Predictive of response to immunotherapy

**Table 2:** Summary of key studies evaluating *p16* and *MTAP* immunohistochemistry in melanocytic lesions.

Study/Marker	Clones	Reported % Cut-Offs/Thresholds (Interpretation)	Histotype/Sensitivity/Specificity/PPV/NPV	Internal and External Controls	Reference
Reed et al., 1995—p16	Clone G175-405 (Pharmingen)	Qualitative evaluation of the presence or absence of nuclear ± cytoplasmic staining. Complete loss is defined as the absence of staining in melanocytes with preserved positivity in non-neoplastic elements.	Benign nevi, dysplastic nevi, Spitz and Reed nevi, primary and metastatic melanomas. Estimated sensitivity ≈ 70%, specificity ≈ 90% for distinguishing melanoma from benign nevus.	Internal: keratinocytes and fibroblasts positive. External: positive control not specified (routine FFPE control blocks).	[31]
Al Dhaybi et al., 2011—p16	CINtec^®^ p16^INK4A^ (E6H4 clone, Ventana)	Qualitative binary system: Positive (retained) = nuclear ± cytoplasmic staining (diffuse or “checkerboard”). Negative (loss) = complete absence in melanocytes with intact internal controls.	Spitzoid melanomas, Spitz nevi, benign nevi (pediatric). All melanomas negative, all nevi positive → Apparent sensitivity = 100%, specificity = 100%, PPV = 100%, NPV = 100% (limited by small sample size).	Internal: keratinocytes, fibroblasts, endothelial cells, and inflammatory cells positive. External: CINtec^®^ positive control tissue (cervical epithelium) and negative control by omission of primary antibody.	[52]
Yazdan et al., 2014—p16	Clone JC8 (Santa Cruz Biotechnology)	Semi-quantitative nuclear scoring: 0 = complete absence, 1 = aggregates with loss, 2 = patchy loss, 3 = retained. Scores 0–1 = negative; 2–3 = positive.	Atypical Spitz tumors with heterozygous vs. homozygous 9p21 deletions. P16 retained in 67% of heterozygous vs. 0% of homozygous cases (*p* = 0.0005).	Positive and negative controls performed for each run (Leica Bond-Max platform).	[49]
Harms et al., 2016—p16	CINtec^®^ p16 INK4A (E6H4, Ventana/Roche)	Two systems used: h-score = intensity (0–3) × % positive cells (0–300). Binary qualitative: Loss = complete/near-complete absence; Retained = any nuclear/cytoplasmic staining.	Spitz nevi, atypical Spitz tumors, spitzoid melanomas. P16 loss in 26% of ASTs and 16% of spitzoid melanomas. Low sensitivity (~16%), high specificity (100%) for malignancy.	Internal: epidermal and dermal cells positive within the same section. External: validated positive FFPE tissue per Ventana protocol.	[53]
He LJ et al., 2025—*p16* and *MTAP*	CINtec^®^ p16 (E6H4, Ventana/Roche); MTAP 2G4D2 (Abnova)	Binary qualitative system: Retained = distinct nuclear ± cytoplasmic (p16) or cytoplasmic (MTAP) staining. Loss = complete absence in melanocytes with intact staining in non-neoplastic cells. “Mosaic” p16 recorded separately.	Melanomas and nevi. For CDKN2A homozygous deletion: p16 → 82%/43%/35%/87%. For *MTAP* loss (*CDKN2A* deletion surrogate): 41%/100%/100%/82%.	Internal: dermal fibroblasts and keratinocytes positive. External: Ventana p16 control tissue; *MTAP*-positive control block.	[46]
Behrmann et al., 2003—MTAP	Anti-MTAP (polyclonal antibody; FFPE, manual staining)	Qualitative binary system: retained vs. complete cytoplasmic loss in melanocytes. Absence of staining interpreted as “loss.”	Primary and metastatic cutaneous melanomas (not specified). Not reported numerically; *MTAP* loss correlated with advanced melanoma stage and tumor progression.	Internal: stromal and epidermal cells positive. External: standard laboratory positive and negative controls.	[36]

## Data Availability

No new data were created or analyzed in this study. Data sharing is not applicable to this article.
